# Acceptability of virtual psychiatric consultations for routine follow-ups post COVID-19 pandemic for people with intellectual disabilities: cross-sectional study

**DOI:** 10.1192/bjo.2024.21

**Published:** 2024-04-19

**Authors:** Samuel Tromans, Sarah Rybczynska-Bunt, Sarah Mitchell, Susan Cummins, David Cox, Jennifer Downing, Paul H. Lee, Lucy Teece, Tony Marson, Rohit Shankar

**Affiliations:** Department of Population Health Sciences, University of Leicester, UK; Adult Learning Disability Service, Leicestershire Partnership NHS Trust, Leicester, UK; Community and Primary Care Research Group, University of Plymouth, UK; Cornwall Intellectual Disability Equitable Research (CIDER) Cornwall Partnership Foundation Trust, Truro, UK; Pharmacology and Therapeutics, University of Liverpool, UK; Southampton Clinical Trials Unit, University of Southampton, UK; Cornwall Intellectual Disability Equitable Research (CIDER) Peninsular Medical School, University of Plymouth, Truro, UK

**Keywords:** COVID-19, developmental disabilities, virtual consultations, remote consultation, risk

## Abstract

**Background:**

After the rapid implementation of digital health services during the COVID-19 pandemic, a paucity of research exists about the suitability of remote consulting in people with intellectual disabilities and their carers, particularly for neuropsychiatric reviews.

**Aim:**

This study examines when remote neuropsychiatric routine consulting is suitable for this population.

**Method:**

A survey was conducted of people with intellectual disabilities and their carers, examining their preference between face-to-face and video consultations for ongoing neuropsychiatric reviews within a rural countywide intellectual disability service in Cornwall, England (population: 538 000). The survey was sent to all adults with intellectual disabilities open to the service on 30 July 2022, closing on 30 September 2022. Participants were asked to provide responses on 11 items predesigned and co-produced between clinicians and experts by experience. The entire service caseload of people had White ethnicity, reflecting the ethnic demographics of Cornwall. Responses received without consent were excluded from the study dataset.

**Results:**

Of 271 eligible participants, 119 responses were received, 104 of whom consented to having their anonymised data used for research analysis. There were no significant differences between preferences and age and gender variables. There was no statistically significant difference regarding preference for the reintroduction of face-to-face appointments (52.0%) compared with video consultations (48.0%). Travel distance (>10 miles) to the clinical setting was important but did not outweigh benefits for those preferring a face-to-face appointment.

**Conclusions:**

This study offers insights into the factors that influence preferences about what type of neuropsychiatric appointment is most suitable for people with intellectual disabilities.

Digital healthcare is part of the National Health Service's (NHS's) long-term plan for a more sustainable and efficient health service.^1^ The rapid implementation of video consulting in the wake of the COVID-19 pandemic has divided opinion, with some patients enjoying its convenience, while others are concerned about it generating inequity in access.^2^ As there is a transition out of the COVID-19 pandemic, there is an increasing focus on how to develop effective hybrid models of access to healthcare that identifies and responds to individuals who experience digital access challenges.

## Virtual consultations in psychiatry

While virtual consultations have been long discussed in psychiatry, and treatment outcomes comparable with those associated with face-to-face consultations have been reported, there are a myriad of technical and ethical issues requiring navigation.^3^ Technical issues include limited visibility of the entire person (impacting on the perceptions of each other's body language), audio disruptions (e.g. voice distortion), lack of a shared spatial environment and technical issues (e.g. internet connectivity). Ethical issues include autonomy (e.g. the individual's increased control over their environment), lucidity (e.g. relating to technical difficulties) and confidentiality (e.g. due to reduced control over the therapeutic space).^3^

A systematic review pertaining to the acceptability to patients of video consultations within mental health services^4^ reported five interacting patient-related factors that promoted acceptability. These included barriers to accessing services in person, a pre-existing trusting relationship with their therapist, technical problems being minor and promptly addressed, a less personal meeting being expected, and less complex difficulties. However, there is a relative lack of randomised controlled trial research relating to video consultation models employed in community mental health settings,^5^ though research is currently being conducted to address this knowledge gap.^5^

## Challenges with people with intellectual disabilities

It is well known that people with intellectual disabilities continue to be subject to poorer health outcomes^6^ and to be underserved and underrepresented in research.^7,8^ The shift to a remote service in the wake of the COVID-19 pandemic has led to questions about the impact on people with intellectual disabilites^9,10^ Ofcom's report into disabled people's access to and use of communication devices and services^11^ found that people with intellectual disabilities have reduced technological capability and digital literacy. Indeed, many rely on support from their carers to facilitate digital inclusion,^12^ rather than being able to engage independently.

## Virtual consultations in psychiatry for people with intellectual disabilities

People with intellectual disabilities have a substantially increased burden of mental and physical health problems relative to their peers without intellectual disability.^13,17^ Walton and colleagues^18^ report a lack of high-quality evidence relating to the organisation, structure and delivery of mental health services for individuals with mild intellectual disability, who represent the majority of people with intellectual disabilities. A systematic review relating to the experiences of adults with intellectual disability accessing digital mental health interventions^19^ concluded that such approaches have potential in providing support to this patient group. However, such a role may be supplementary to face-to-face consultations, rather than as a substitute for them.^19^ A scoping review specifically related to virtual consultations for adults with intellectual and developmental disabilities^20^ concluded that ‘it is possible to deliver accessible, high quality virtual care’ for this patient group, though it did cite the lack of research on this topic and expressed a need to understand more about the characteristics of patients for whom virtual care can be successfully delivered. In their rapid review, another study^12^ focused exclusively on the digital inclusion and participation of people with intellectual disabilities during the COVID-19 pandemic, reporting that digital exclusion is a significant issue for this patient group, with access to relevant digital devices and availability location and support in using them presenting significant barriers. Paid carers involved in the direct support of people with intellectual disabilities for both virtual and in-person appointments describe positive and negative experiences with both modalities.^21^ Furthermore, a qualitative study pertaining to video consultations in primary care for adults with intellectual and developmental disabilities,^22^ involving semi-structured interviews of patients, carers, support staff, and physicians, concluded that there was a need for flexibility with the modalities by which care was delivered, and that the option of virtual consultations helped improve access.

Beyond concerns about technological capability is how healthcare services, particularly psychiatric services, make decisions on when video is or is not an appropriate modality to carry out a consultation with an individual with intellectual disabilities. Less is known about how video consulting is experienced by people with intellectual disabilities, as well as the potential clinical risks and potential for clinical diagnoses to be missed relative to face-to-face consultations. If video consulting continues to be adopted and scaled up in psychiatric circles, it is important we understand the needs and preferences of people with intellectual disabilities, while keeping in mind the vast heterogeneity in the intellectual disability population.

## Aims

- To test out the acceptability of video consulting among people with intellectual disabilities and their carers
- To measure correlations between preferences and several key variables, including age, gender and travel distance to the clinical setting

## Method

A cross-sectional survey using the STrengthening the Reporting of OBservational studies in Epidemiology (STROBE) guidance to report was designed (Supplementary information 1 available at https://doi.org/10.1192/bjo.2024.21). The study population were people with intellectual disabilities attending sessions with neuropsychiatrists in an adult intellectual disability service in an economically poor rural part of the south-west UK (Cornwall, UK, population: 538 000). The study population were all open to the services, and no new patients (those for first assessment) were included. A bespoke structured questionnaire was designed by a group of clinical experts and CHAMPS (the Cornwall Intellectual Disability and Autism Support team, i.e. seven people with intellectual disabilities and/or autistic spectrum conditions) who are a group of experts by experience from the county council (Supplementary information 2 and 3). The local intellectual disability service consultants and epilepsy nurses reviewed the survey from a clinical perspective.

People with intellectual disabilities open to the psychiatric caseload of the service were contacted to complete a descriptive postal survey on their preferences for video or face-to-face consultations. The survey was designed to take approximately 8–10 min to complete. This was felt to be the optimum time to balance response engagement and gain the minimum required information to draw meaningful conclusions. The survey had a mixed methods approach consisting of questions with 11 predetermined answers and questions that allowed for free text comments. Of the two sections of pre-determined choices (Supplementary information 4), the first section had seven choices to ascertain why video consultation is preferred. The second section had four options on what face-to-face review was preferred. The participants could select all options which seemed relevant to them. They were also asked additional questions to identify any technological problems they had with devices, software and connectivity, and digital support to access their video appointment. The survey met the NHS Accessible Information Standard.^23^ Participants were invited to identify whether they had help in completing it and who supported them to do so; the options ranged from: a carer looking after client at home, relative with family member in residential setting, support worker within a care team, team lead or manager of a care team, or other. No incentive was offered.

Participants were eligible to take part if they were adults (defined as being over 18 years of age) and registered on the Trust system as having an intellectual disability and being open to a psychiatrist. Participant responses were considered in terms of younger (<40 years) and older (≥40 years) age groups; these age grouping were determined in accordance with the IDS-TILDA study (Intellectual Disability Supplement to the Irish longitudinal study on ageing), which defined older persons with an intellectual disability as being aged 40 years or older, to reflect their reduced life expectancy relative to the general population.^24^ There were no exclusion criteria around mental capacity, and clients with both mild and moderate-profound intellectual disability were invited to take part. The surveys were distributed across all mental health teams across the county. The surveys were sent out on 30 July 2022 with the survey closing on 30 September 2022. Of the responses received, data were utilised only from respondents who consented to their anonymised data being used for academic analysis and research publication. Responses that were received without such consent were excluded from the study dataset.

### Ethics

The study was compliant with the Declaration of Helsinki.^25^ No formal ethics were needed for this project; this was determined via the NHS Health Research Authority tool, which determined that the study would not be considered research by the NHS as it does not involve either participant randomisation or deviation from accepted standards of treatment, and the findings are non-generalisable in nature (Supplementary information 5). The survey was registered as a service evaluation at the local NHS Trust. All participants were advised at the start of the study that participation was voluntary and that, if they chose to participate, their replies would be anonymised and analysed. Data were pooled prior to analysis.

### Analysis

Descriptive statistical analyses were carried out using Microsoft Excel. Differences in acceptability were assessed using Fisher's exact test for those who preferred video consultations, those that did not mind either or those who opted for in-person consultations. Respondents who selected ‘both video and face to face’ were incorporated into both the video and face-to-face options. Thus, some respondents were double counted, but equally in both groups. Preferences were compared against the distributions of age, gender and distance of residence from clinic. Significance was accepted at *P* < 0.05 where tested.

## Results

The population surveyed consisted of the total caseload of all psychiatrists working in the specialist neuropsychiatric intellectual disability service in the county: 271 patients. A total of 119 responses were received, of which 104 people (38.3%) consented to having their anonymised data used for research analysis. Of these 104, there were 54 (51.9%) individuals aged less than 40 years, and 50 (48.1%) individuals aged 40–74 years. Sixty-eight were male (65.4%), and 36 (34.6%) were female. Of all replies, 12 (11.6%) forms were completed only by family members or carers representing the client, 37 (35.6%) forms were completed with clients by family members caring for the client at home, 25 (24.0%) supported by a care team manager and 23 (22.1%) aided by a care team support worker. Just 4 (3.8%) of the forms were completed only by the patient. Three (2.9%) respondents did not answer this question.

Twenty-three participants (22.1%) reported that both video and face to face were acceptable to them, while 81 (77.9%) reported that only one of these two consultation modalities was acceptable. Of 127 responses (23 for duplicates in both group) included in the analysis, 61 responses (48%) expressed a preference for video consultations, and 66 responses (52%) expressed a preference for face-to-face consultation.

### Relationship between demographic variables and consultation preferences

Just over half of participants (54.3%) (*n* = 38/70) in the younger age group (20–39 years) selected video review. Three out of five participants in the older age group (40 years and over) selected face-to-face review (59.6%) (*n* = 34/57 in older age group). More females selected video consultation (57.8%) (*n* = 26/45 females). Likewise, more males selected face-to-face consultation (57.3%) (*n* = 47/82 males). There was no significant difference between preferences but there was suggestiveness of trends. Of all respondents, 51.0% (*n* = 64/127) were willing to travel more than 10 miles for a review, while 42/127 (33%) were not and 16% (*n* = 21/127) did not reply one way or the other.

### Reasons for virtual consultation

Table 1 shows factors that influence the acceptability of video consultations for ongoing care and treatment. Those people that preferred video consulting were significantly more likely to select the ‘This is the best option for me in terms of travelling and potential unfamiliar locations’ response option, compared with respondents who stated a preference for face-to-face reviews (*P* = 0.006). Virtual consultation supporters were also significantly more likely to select the ‘I can be at home in comfort’ response option as compared to face-to-face preferrers (*P* = 0.018). A major factor for those individuals who expressed preference for face-to-face consultation (Table 2) was a need to be physically present in the same room as the psychiatrist, as compared to those preferring virtual consultations (*p* = 0.016).

**Table 1:** Factors that influence the acceptability of video-reviews

Reason for video preference	Video preference	Face-to-face preference	Total	Fisher's exact *P*-value
I don't have to travel	28	14	42	0.286
I can be at home in comfort	37	15	52	0.018
I find it easier to see the consultant by video than being in the same room as them	4	2	6	1.00
My family can join the review when I do not live with them and live away from me	18	11	29	0.822
My care team don't have to worry about people being ill or on leave to get me to the appointment	13	8	21	1.00
This is the best option for me in terms of travelling and potential unfamiliar locations	18	3	21	0.006
I live with my family and it saves on time and travelling for us	20	11	31	0.653

**Table 2:** Factors that influence the acceptability of face-to-face reviews

Reason for face-to-face preference	Video preference	Face-to-face	Total	Fisher's exact *P*-value
I like to be in the same room as people	19	46	65	0.016
I like to go out from my house and have a drive	15	30	45	0.517
We do not have technology to join video reviews	0	6	6	0.080
We do have technology but we are not confident in using it	0	3	3	0.290

## Discussion

This paper is one of the first papers which generates important insights into a remote service offering opportunities for a more effective and convenient way of consulting for people with intellectual disabilities, and when it potentially creates risk and inequity of access. The varied attitudes to remote consultations from a patient survey indicate a need for a more flexible and hybrid health service.

### Limitations

The service surveyed a small sample of people with intellectual disabilities, and as carers were heavily involved in supporting respondents to complete the form, their own views could have potentially influenced the patients’ responses. Consequently, there has been no attempt to stratify the population based on their health conditions, to understand the nuances (such as level of intellectual disabilities, and co-occurring conditions such as autism, etc.) that feed into the preferences for video or in-person health appointments. Very little is known on how the type of device (mobile phone, tablet, computer) or quality of the video and/or audio used by individuals affects their attitudes towards remote consulting. The paper does not shed light on several considerations when deciding on the appropriateness of a consulting modality, which include: whether it is clinically appropriate (i.e. the individual's neuropsychiatric condition), whether the individual finds remote modalities acceptable even if their preference is face to face, and when it might improve or hinder access to healthcare. Other confounders include the role of socioeconomic deprivation and ethnicity considerations.

Furthermore, it is difficult to reliably determine the level of influence of carer views in the reported findings, particularly among participants with more severe intellectual disability and/or communication impairment. Additionally, we were unable to report data on the characteristics of the background patient population from which the study population were sampled (other than with respect to ethnicity), and thus it is unclear whether the study population are representative of this larger group. In relation to ethnicity, the people in the entire study population were White; whilst this is not markedly dissimilar from the wider population of Cornwall, where 2021 census data indicate that 96.8% respondents were White,^26^ it means that the study's findings are not representative of non-White ethnic groups. Additionally, we did not collect data pertaining to the history of consultation modalities of individual patients (i.e. having been seen solely virtually, solely in person, and reviewed via both modalities); collecting such data would be valuable in the context of future research in this area. We also did not collect data pertaining to participants’ care provision (whether they received support from family or paid carers); further research could evaluate whether such factors influence participant responses. Finally, certain question items on the survey could have been phrased more neutrally; the survey was co-developed with experts by experience, and this suggests a need for further training pertaining to risk of bias and leading questions.^27^

### Implications for research

More work needs to be done to identify preferences in a larger sample of people with intellectual disabilities and to stratify the population to understand the factors that shape patient preferences. This will involve thinking about how risk presentations or intellectual disability subgroups affect decisions on the type of appointment needed. For example, male patients are more likely than female to be prescribed stronger psychotropic medication to help manage mood and maladaptive behaviours. Although not statistically significant, in our patient survey the greater preference was for in-person consultations amongst male respondents. Similarly, there are issues of whether family or professional carers seek more face-to-face consultation, and why.

An economic evaluation is also needed to understand whether remote consultations are really effective and efficient compared with standard care for this population. Health service efficiencies will be explored against patient health outcomes and the potential for over-investigation, missed diagnoses and over-prescribing. Further research should explore risk, not only in terms of patient safety but also indemnity risks to the NHS and how organisational risk is managed. Furthermore, it would be valuable to explore how the views of people with intellectual disabilities and their carers compare, identifying important similarities and differences. Additionally, further study needs to be undertaken of the safeguarding risks associated with virtual consultations, as people with intellectual disabilities represent a group particularly vulnerable to abuse,^28^ and a virtual environment potentially enables easier involvement of individuals not acting in the patients’ best interests in the clinical consultation.^29^ While challenges exist to engage people with intellectual disabilities in research, there is an appetite both from those with disabilities and researchers to have meaningful engagement.^27^

### Implications for practice

The role of digital appointments should be to augment, not replace, all face-to-face provision and health services. As suggested by Lunsky et al,^21^ ‘optimal care depends on maximizing the fit between the person's abilities, the skill set of direct support professionals and health care providers, and the presenting health care issue.’ Indeed, Selick et al^22^ report situations whereby virtual technology substantially improved access to healthcare for patients with intellectual and developmental disabilities. Healthcare providers should do benefit and risk calculations before offering remote appointments, but may benefit from a decision-making tool to ensure that patients are offered the most appropriate consultation type (phone, video, in-person or home visit) according to their needs.

### Implications for policy

While increased digitalisation is part of the NHS strategy for a more efficient health service, it is widely recognised that some individuals will require non-digital alternatives. Health settings would benefit from policy guidance around the sorts of reasonable adjustments they need to make to offer a flexible service and to meet patient need and offer non-digital alternative pathways. Furthermore, the role social care plays in organising digital and non-digital appointments needs to be thought about in the context of integrated care systems (ICS), defined by NHS England as ‘partnerships of organisations that come together to plan and deliver joined up health and care services, and to improve the lives of people who live and work in their area’.^27^ Thus, ICS need to be sensitive to the specific needs of people with intellectual disabilities within their region, ensuring that the care provided is of a high standard, equitable and financially responsible.^30^ As services are increasingly delivered remotely, the digital competencies of support workers become more important. This may involve investment into opportunities for digital upskilling, as well as the design of accessible video consulting software to ensure familiarisation with one interface across health and social care services.

## Supporting information

Tromans et al. supplementary material 1 Tromans et al. supplementary material

Tromans et al. supplementary material 2 Tromans et al. supplementary material

Tromans et al. supplementary material 3 Tromans et al. supplementary material

Tromans et al. supplementary material 4 Tromans et al. supplementary material

Tromans et al. supplementary material 5 Tromans et al. supplementary material

## Data Availability

The data that support the findings of this study are available from the corresponding author upon reasonable request.
