# Supplementary material for: Acceptability of virtual psychiatric consultations for routine follow-ups post COVID-19 pandemic for people with intellectual disabilities: cross-sectional study
Source: BJPsych Open. 2024 Apr 19;10(3):e90. doi: 10.1192/bjo.2024.21 (PMC11060089; doi:10.1192/bjo.2024.21)
Supplement: Tromans et al. supplementary material 2 — Tromans et al. supplementary material [file S2056472424000218sup002.pdf]

Cornwall Partnership
NHS Foundation Trust

Future Reviews with the Learning Disability Consultants

1. **What is your relationship to our client?**
   - ☐ Our client who sees the consultant
   - ☐ Relative caring for family member at home
   - ☐ Relative with family member in a residential setting
   - ☐ Support worker within a care team
   - ☐ Team Lead / Manager of care team / residential setting
   - ☐ Other (please say……………………………………………………………………)

2. **What area of the county does our client live in?**
   - ☐ Penwith
   - ☐ Kerrier
   - ☐ Carrick
   - ☐ Restormel
   - ☐ Caradon
   - ☐ North Cornwall

3. **How do you want to see your consultant for your next review?**
   - ☐ by video
   - ☐ face to face

4. **Why do you like to see them by video? (select as many as appropriate)**
   - ☐ I don't have to travel
   - ☐ I can be at home in comfort
   - ☐ I find it easier to see the consultant by video than being in the same room as them
   - ☐ My family can join the review when I do not live with them and live away from me
   - ☐ My care team don't have to worry about people being ill or on leave to get me to the appointment
   - ☐ This is the best option for me in terms of travelling and potential unfamiliar locations
   - ☐ I live with my family and it saves on time and travelling for us

5. **Why do you like to see the consultant face to face? (select as many as appropriate)**
   - ☐ I like to be in the same room as people
   - ☐ I like to go out from my house and have a drive
   - ☐ We do not have technology to join video reviews
   - ☐ We do have technology but we are not confident in using it
   - ☐ **Please tick if you would like to have a 1:1 chat to help you to be able to do this with one of the medical secretaries**

6. **While we do our best to locate clinic rooms near to you, they may not always be available with current restrictions or not as large as we would like. How far would you travel to enable a face to face review?**
   - ☐ Up to 5 miles
   - ☐ Up to 10 miles
   - ☐ Up to 20 miles
   - ☐ More than 20 miles

7. We have used the venues below for clinics pre lockdown though this might change. If you would rather be seen in clinic face to face which of the venues below would you prefer? You can choose more than one.

- ☐ Bodmin Clinic
- ☐ Launceston Medical Centre
- ☐ Chy Govenek, Threemilestone
- ☐ Camborne & Redruth Hospital (Barncoose) Outpatients
- ☐ Basset Unit, Camborne & Redruth Hospital
- ☐ John Daniel Centre, Penzance
- ☐ Other – please state where …………………………………………………..

Any comments:

8. **Did you join a video review with us in the past year?** Yes No

9. If you had a problem joining a video review with us in the past year, can you tell us why?

- ☐ Poor internet connection (yours)
- ☐ Poor internet connection (ours)
- ☐ Accessing links as they were not clear to join
- ☐ Lack of supporting information on how to join
- ☐ Do not own a device to join a video review
- ☐ Did not have any problems – happy with how things were enabled

Something else? (please comment)

10. How have you found your communication with the LD Medical & Epilepsy Team during the last year with COVID restrictions compared to before?

- ☐ Extremely effective
- ☐ Very effective
- ☐ About the same
- ☐ Less effective
- ☐ Not at all effective

Any additional comments as to why you chose the above option? i.e what you liked and didn't like

11. With reviews by video were you happy with the notice of clinic appointments, speed of receiving clinic letters via email, ability and ease of contacting consultants, medical secretaries, epilepsy nurses during the lockdown period

- ☐ Happy with notice of appointments before they took place
- ☐ Unhappy with notice of appointments before they took place
- ☐ Happy with speed of clinic letter delivery via email (within 5 working days if not sooner)
- ☐ Unhappy with speed of clinic letter delivery (more than 10 days)
- ☐ Found it easy to contact the team via email
- ☐ Quick response when you contacted the medical secretary to speak to nurses / gain info from consultants

Any additional comments

☐ Please tick here if you **do not** wish to share your answers above which will be anonymised with Plymouth University as part of a wider national study as to using video platforms during lockdown

Cornwall Partnership
NHS Foundation Trust

Getting in touch with you

We want to make sure your contact details are correct

**Client Name:**			
**Who is the best person to contact to arrange your review?**			
**Name**	**Phone Number**	**Email**	**Relationship to you**
**Is there anyone else we should ask to join you in your review like a family member who does not live with you, an advocate?**			
**Name**	**Phone Number**	**Email**	**Relationship to you**

Review days

Reviews are usually held on Mondays. Tuesdays or Thursday. Which day would work best for you subject to activities you already attend such as days with support staff, college days or work days?		
☐ Monday	☐ Tuesday	☐ Thursday

Review times

You might not be an early riser, you might be at college all day or at an activity centre and there might be times we should avoid when arranging your review. Which of the time slots below would be good for you – select as many as you want:			
Between<br>☐ 09.30 – 10.30	Between<br>☐ 10.30 – 12.30	Between<br>☐ 13.00 – 15.00	Between<br>☐ 15.00 – 16.30

If you have any questions about your review please contact Sarah Mitchell by phone or email:
Email: sarah.mitchell20@nhs.net Telephone: 01872 221551

Thank you in advance for completing and returning this. Your thoughts are important in helping us in planning your individual and personal health review.
