# Supplementary material for: Acceptability of virtual psychiatric consultations for routine follow-ups post COVID-19 pandemic for people with intellectual disabilities: cross-sectional study
Source: BJPsych Open. 2024 Apr 19;10(3):e90. doi: 10.1192/bjo.2024.21 (PMC11060089; doi:10.1192/bjo.2024.21)
Supplement: Tromans et al. supplementary material 3 — Tromans et al. supplementary material [file S2056472424000218sup003.pdf]

Your Name: ___________________________

NHS
Cornwall Partnership
NHS Foundation Trust

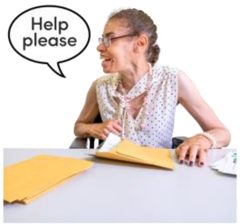	Can you help us
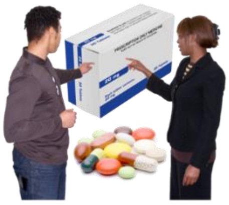	We want to ask you questions about lockdown and seeing your doctor and nurse for your medication review
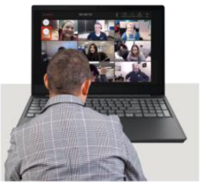	If you liked seeing them on a computer or tablet
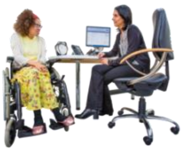 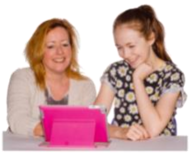	How you want to see them when lockdown is over
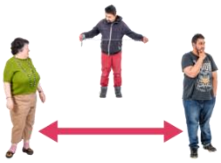<br>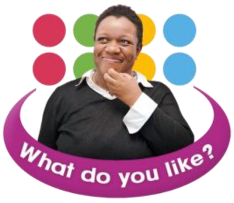	We want to do what is best for you and to keep everyone safe.<br><br>We want you to tell us what suits you to see your doctor next time

Who is your doctor? Draw a circle around the one you see

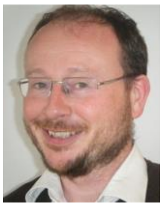 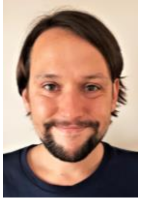 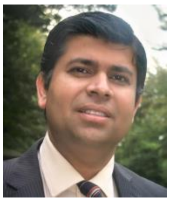 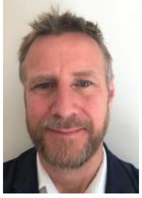

Who is your nurse? Draw a circle around the one you see

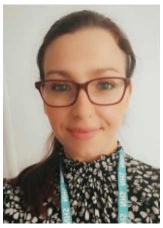 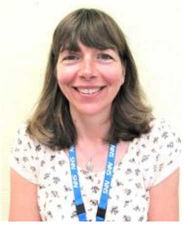 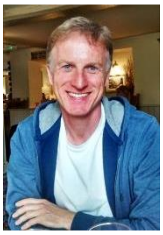 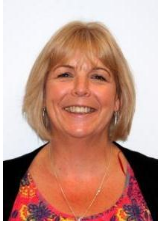 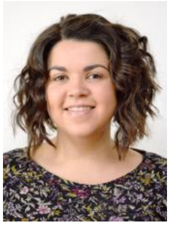 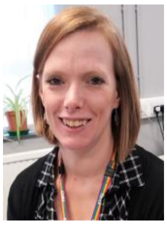 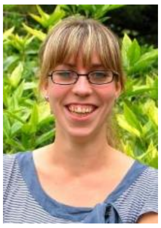

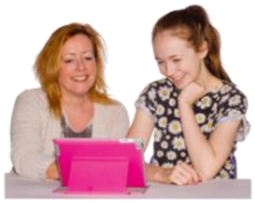	During lockdown how was using a video to see the doctor and nurse and not face to face<br>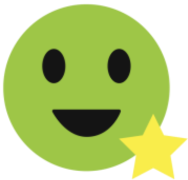 Very good 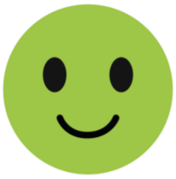 Same as before 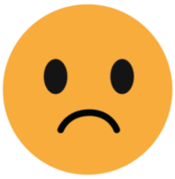 Did not like it
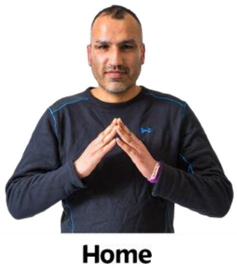	I want to keep seeing the doctor and nurse from my home by video?<br>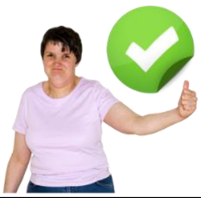 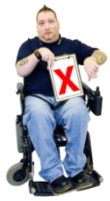 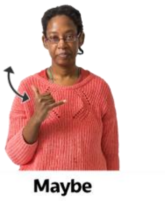
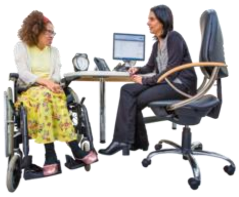	I want to see the doctor and nurse face to face as it makes me happier when we can<br>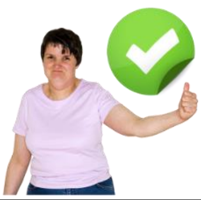 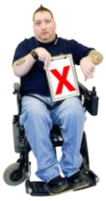
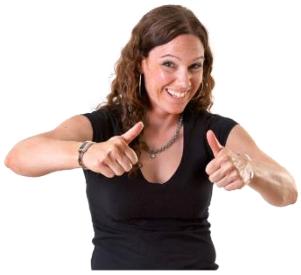	I would be happy to see the doctor and nurse both<br>face to face 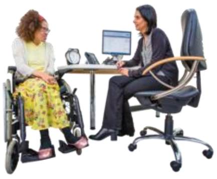 video 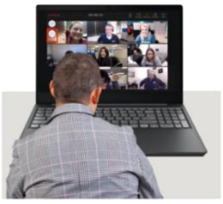
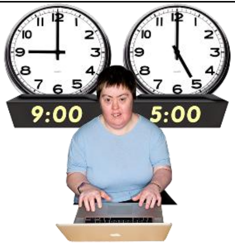	When do you like to see your doctor<br>in the morning 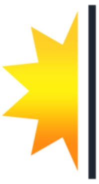 or in the afternoon? 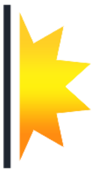
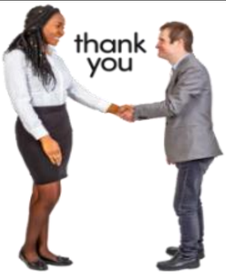	Thank you for helping us
