# Supplementary material for: Acceptability of virtual psychiatric consultations for routine follow-ups post COVID-19 pandemic for people with intellectual disabilities: cross-sectional study
Source: BJPsych Open. 2024 Apr 19;10(3):e90. doi: 10.1192/bjo.2024.21 (PMC11060089; doi:10.1192/bjo.2024.21)
Supplement: Tromans et al. supplementary material 4 — Tromans et al. supplementary material [file S2056472424000218sup004.docx]

Table 1: List of pre-selected options for preferring video or face-to-face appointments

| Why do you like to see them by video? | - I don’t have to travel - I can be at home in comfort - I find it easier to see the consultant than being in the same room as them - My family can join the review when I do not live with them and live away from me - My care team don’t have to worry about people being ill or on leave to get me to the appointment - This is the best option for me in terms of travelling and potential unfamiliar locations - I live with my family and it saves on time and travelling for us |
| --- | --- |
| Why do you like to see the psychiatrist face to face? | - I like to be in the same room as people - I like to go out from my house and have a drive - We do not have technology to join video reviews - We do have technology but we are not confident in using it |
